# Anatomical variation in the cervical branch of the facial nerve: a systematic review

**DOI:** 10.1007/s00276-026-03969-w

**Published:** 2026-10-12

**Authors:** Joseph Pathiparampil Mano, Shubham Dave, Mohammed Faraaz Saiyed, Yangmyung Ma, Kallirroi Tzafetta, Sami Al-Ani

**Affiliations:** 1 University of Birmingham, Birmingham, UK; 2 University Hospitals Birmingham NHS Foundation Trust, Birmingham, UK; 3 Imperial College London, London, UK; 4 St Andrew’s Centre of Plastic Surgery & Burns, Broomfield Hospital, Chelmsford, UK; 5 Aston Medical School, Birmingham, UK

**Keywords:** Anatomy, Cervical branch, Facial nerve, Variation, Platysma, Branching pattern, Neck surgery, Neck dissection

## Abstract

**Background:**

There is a paucity of literature on the anatomical variations of the cervical branch of the facial nerve (CBFN). Poor awareness of its anatomy and variations risks intraoperative damage, misdiagnosis of facial nerve injury and undesirable functional and aesthetic outcomes for patients.

**Purpose:**

To review anatomical studies on the CBFN and synthesise data on its course, innervations and common variations.

**Methods:**

A PRISMA-compliant systematic review of anatomical studies of the cervical branch of the facial nerve was conducted. PubMed, Embase, Scopus, Cochrane Library, Web of Science, and Google Scholar were searched up to July 2025. Papers were quality appraised, and data relevant to the anatomy of the CBFN were extracted.

**Results:**

Thirty-five papers with a collated sample size of 867 hemi-necks were identified: 25 with anatomical dissections and 10 case reports. The mean number of CBFN branches was 1.34 (sensitivity range 1.31–1.36). Communication with the marginal mandibular nerve, transverse cervical nerve and great auricular nerve was variable, with prevalences ranging from 22—97%. Distances between the nerve and anatomical landmarks were heterogeneous. Many individual cases of cervical branch variation in course and relationship to nearby vasculature were identified.

**Conclusion:**

The cervical branch of the facial nerve displays wide variability in its course. Pre-operative mapping, careful dissection and intra-operative nerve stimulation to aid accurate identification are recommended to prevent iatrogenic damage.

## Introduction

The facial nerve (CN VII), through its motor and sensory function, is key to facial expression and effective communication. Within the parotid gland, the facial nerve gives rise to its five motor branches, which extend across the hemi-face and neck: temporal, zygomatic, buccal, marginal mandibular, and cervical [7]. While much has been written about the anatomy of other branches of the facial nerve, especially the marginal mandibular branch (MMB), an area that has been relatively overlooked is the anatomy of the cervical branch of the facial nerve (CBFN), possibly due to underappreciation of complications from its injury [7].

An understanding of the position and route of the CBFN is primarily derived from descriptions in anatomy textbooks, such as Gray’s Anatomy, and supplemented by findings from cadaveric studies or a clinician’s own practice; of the cervical branch, Gray’s Anatomy describes: *The cervical branch emerges from the lower part of the parotid gland and runs anteroinferiorly under platysma to the anterior neck. Typically single, it supplies platysma and communicates with the transverse cutaneous cervical nerve* [45].

Acland’s Atlas of Human Anatomy provides further detail on the CBFN [1], whereby the CBFN is described as a nerve that typically runs closely posterior to the angle of mandible (AoM) after arising from the cervicofacial nerve trunk following its division from the marginal mandibular nerve [9]. From here, the nerve enters the superficial anterior neck, where it carries out its role of providing principle motor innervation to the platysma [8] and contributes to motor innervation of depressor labii inferioris (DLI) [27], a muscle which, at its origin at the oblique line of the mandible, is continuous with the platysma and works with other muscles to depress the lower lip [23]. However, while this is the anatomy described in textbooks, there is a paucity of literature on how its anatomy varies across different ethnicities, age demographics, and sexes.

The platysma often requires significant caution from surgeons as its dissection is often required to access deeper planes, and inattention may lead to direct injury to the platysma or to its innervating nerve, the CBFN. From an aesthetic surgery perspective, the platysma is of particular significance; loss of muscle tone leads to the effacement of neck bands. While this may be normal as individuals age, in instances where surgeries result in iatrogenic injuries to the CBFN, this process may be prematurely triggered, leading to platysma dyskinesia or “turkey neck” [34]. The DLI, which also receives motor supply from the MMB, is one of the depressors of the lower lip and may give rise to the first symptoms following CBFN injury. This may be transient or permanent inability to evert and depress the lower lip, which has been described as a common presentation following iatrogenic injury to CBFN. While typically self-resolving, in rare cases, the effects may be long-lasting, leading to functional difficulties [21].

The neck and lower face are key anatomical regions for a range of surgical specialities and procedures, from otorhinolaryngology surgeons performing parotid and lymph node surgeries to plastic surgeons performing neck lifts and other aesthetic procedures. Thus, CBFN injury is a known complication of several common surgeries, for example, with the estimated incidence of iatrogenic injury in facelift surgery being 1.7% [11]. Therefore, good understanding of the anatomy of the region, as well as evidence-based awareness of anatomical variation across sexes, ethnicities, and age demographics, is key to avoiding iatrogenic injury to the CBFN. This systematic review aims to explore the literature for primary data on the anatomy of the CBFN and identify common anatomical variations to reduce iatrogenic injury, thereby avoiding functional and aesthetic complications for patients.

## Methodology

### Search strategy and eligibility criteria

Searches were conducted in June 2024 across a range of databases, including PubMed, Embase, Scopus, Cochrane Library, Web of Science, and Google Scholar. A further search was conducted using the same strategy in July 2025 to ensure updated results following commencement of the initial review. No date restrictions were imposed in the search. This review was registered on Prospero: CRD420251140960.

The search strategy (Table 1) was created using the population, intervention, comparator, outcome (PICO) framework and included terms such as ‘cervical branch’, ‘dissection’ and ‘anatomic variation.’ Inclusion and exclusion criteria were developed prior to searching. Anatomical studies (comprising either cadaveric or in-vivo dissection), case reports, and case series with primary qualitative or quantitative data on the CBFN were included. Non-English papers, animal studies, and papers not providing primary data on the cervical branch of the facial nerve were excluded. Textbooks and reviews describing the typical course of the CBFN without reporting primary data were excluded.

**Table 1:** Search strategy

("Cervical branch"[All Fields] OR "Cervical nerve"[All Fields] OR "Platysma"[All Fields]) AND ("Anatomy and Histology"[Subheading] OR "Anatomy" [MeSH Terms] OR "Anatomy"[All Fields] OR "Dissection"[MeSH Terms] OR "Dissection"[All Fields] OR "Anatomic variation"[MeSH Terms] OR "Anatomic variation" [All Fields] OR ("Anatomic"[All Fields] AND "variation"[All Fields]) OR "Branches" [All Fields] OR "Variation*" [All Fields] OR "course" [All Fields] OR "Patterns" [All Fields] OR "innervation"[Subheading] OR "innervation"[All Fields] OR ("nerve"[All Fields] AND "supply"[All Fields]) OR "nerve supply"[All Fields])	

### Screening and data extraction

Duplicate results were excluded, and all papers were initially screened by their title and abstract and subsequently by their full text by two independent reviewers, following the Preferred Reporting Items for Systematic reviews and Meta-Analyses (PRISMA) process [35]. Any conflicts were raised in a meeting and were discussed with a third reviewer who was responsible for the final decision. References of papers included were also screened to assess for inclusion. Data extracted from the papers using a bespoke template included: age, sex, ethnicity, syndromic vs non-syndromic, country of study, number of hemifaces, observations on anatomy of CBFN and quantitative data measurements relevant to CBFN, including number of branches and communications. Additionally, a free-text extraction column was included for relevant observational data and descriptions. The full extraction template is available in Appendix Table 8.

### Quality assessment

Papers were quality appraised based on study design. All cadaveric anatomical studies were appraised using the Quality Appraisal for Cadaveric Studies (QUACS) scale [48], a validated tool for quality appraisal for cadaveric studies. For case reports and case series, the Joanna Briggs Institute (JBI) Checklist for Case Reports was used as a validated quality appraisal tool [3]. Two reviewers independently assessed quality using the appropriate tool, and scores were compared to identify any inter-reviewer variation.

### Analysis

Quantitative data were extracted and collated where comparable measures were reported. Observational and qualitative data were thematically analysed. Any deviation from the course of the CBFN compared to the described course in Gray’s Anatomy (43rd edition) was considered as a “variation” [45].

## Results

### Search results

**Fig. 1:**
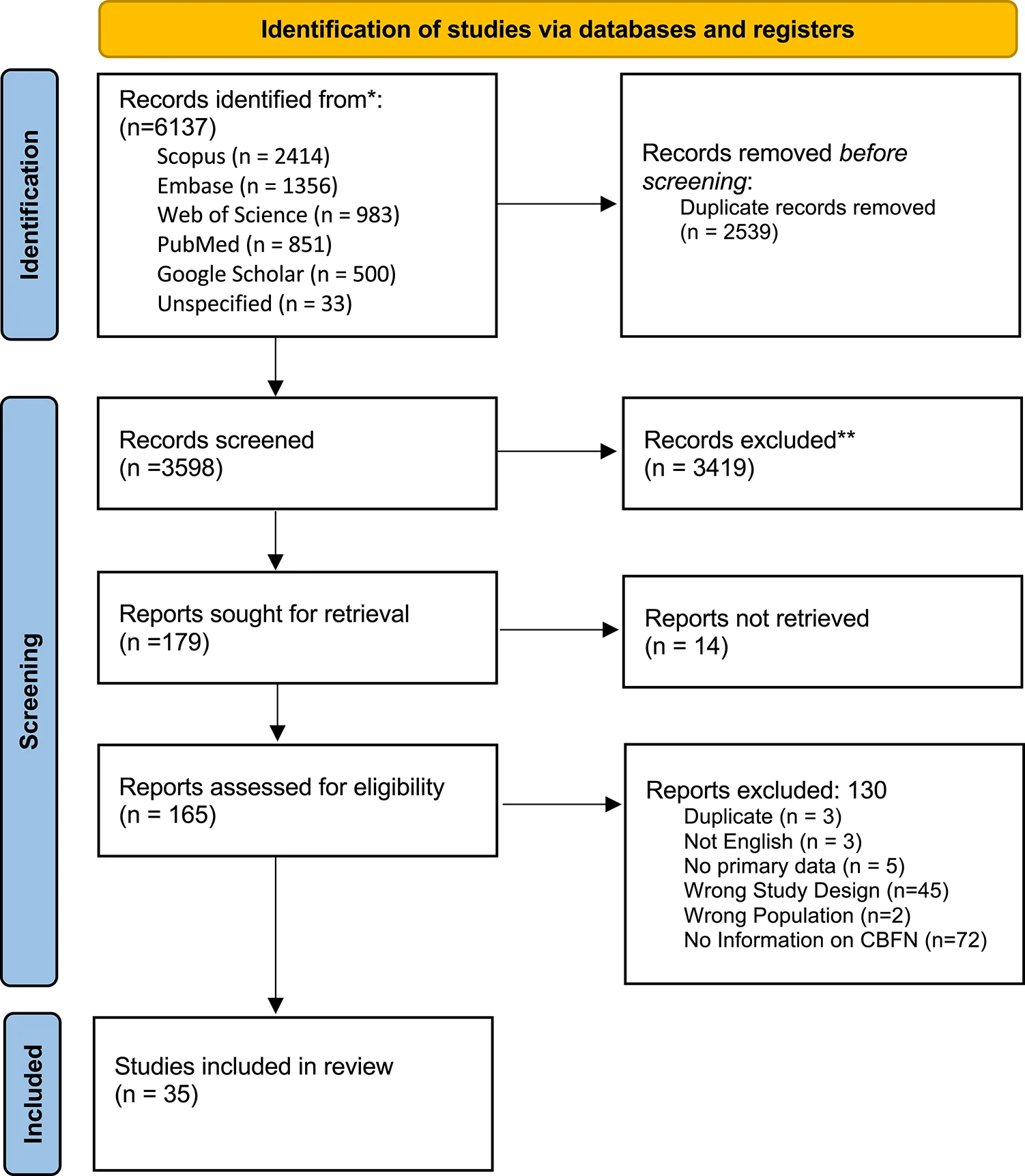
PRISMA diagram of included studies [35]

Searches yielded 6,137 papers, of which 3,243 non-duplicate papers were eligible for screening. After screening against the predetermined criteria, 35 papers were included for review. The selection process is shown in a PRISMA flow diagram in Fig. 1.

Of the 35 papers included for review, 25 were cadaveric/in-vivo dissections, and 10 were case reports/case series. Studies were published across five continents between 1981 and 2024. Across the dissections, data from 867 hemi-necks and 70 isolated parotid glands were reported, with wide heterogeneity in the level of demographic detail reported by each paper. Table 2 shows an overview of all papers included in this review.

**Table 2:** Overview and demographic data of included studies

Author, year	Country	Study design	Hemi—necks (n)	Male: female (n:n)
Al Shaikh et al. [2]	Saudi Arabia	Cadaver dissection	20	NR
Arakawa et al. [4]	Japan	Case report	1	1:0
Babuci et al. [5]	Moldova	Cadaver dissection	75	59:16
Cakmak et al. [7]	Turkey	Cadaver dissection	20	10:10
Chowdhry et al. [9]	USA	Cadaver dissection	16	NR
Cvetko et al. [10]	Japan	Case report	1	0:1
De Bonnecaze et al. [12]	France	Cadaveric and in vivo dissection	30	18:12
Domet et al. [13]	USA	Cadaver dissection	22	NR
Eaton et al. [14]	USA	Cadaver dissection	70 parotid glands	39:31
Farahvash et al. [16]	Iran	Cadaver dissection	42	NR
Freed et al. [17]	USA	Cadaver dissection	40	NR
Gerard et al. [18]	USA	Case report	1	0:1
Gulati et al. [20]	India	Case report	1	NR
Hontanilla et al. [22]	Spain	Cadaveric dissection	12	NR
Jovanovski et al. [25]	Slovenia	Case report	1	0:1
Karakousis et al. [26]	USA	Case report	1	1:0
Kaufman-Goldberg et al. [27]	USA	Cadaveric and in vivo dissection	60	52:8
Klosterman et al. [28]	USA	Case report	2	0:2
Leong et al. [29]	Singapore	Case report	1	0:1
Lindsey et al. [30]	USA, Italy	Cadaver dissection	15	NR
Malins et al. [31]	UK	Cadaver dissection	11	6:5
Martinez Pascual et al. [32]	Spain	Cadaver dissection	38	17:21
Minelli et al. [33]	Australia	Cadaver dissection	12	NR
Rodriguez Lorenzo et al. [36]	Austria	Cadaver dissection	19	NR
Rossell Perry et al. [37]	Peru	Cadaver dissection	64	50:14
Salinas et al. [38]	USA	Cadaver dissection	20	NR
Sinno et al. [40]	USA	Cadaver dissection	16	8:8
Sirasanagandla et al. [41]	India	Case deport	1	1:0
Sirasanagandla et al. [42]	India	Case deport	1	1:0
Socolovsky et al. [43]	Argentina	Cadaver dissection	10	NR
Suganya et al. [46]	India	Cadaver dissection	50	NR
Tzafetta et al. [47]	USA	Cadaveric dissection	10	NR
Woltmann et al. [49]	Brazil	Cadaver dissection	45	NR
Ziarah et al. [50]	UK	Cadaver dissection	110	29:26
Ziarah et al. [51]	Same cohort as the above row*			

NR  Not reported, UK  United Kingdom, USA  United States of America. *Ziarah et al. published two papers reporting data from the same cohort of cadaveric specimen [50, 51]

### Quality assessment

Quality assessment using QUACS [21] resulted in papers scoring between 25 and 92% with a median of 65% (IQR = 17%). Inter-reviewer variation was minimal (1 point per theme). Common reasons for low scores included not reporting the education level of dissecting researchers, findings being observed by only one researcher, absence of or insufficient statistical analysis, and poor declaration of limitations. Basic demographic data for cadavers were also absent in a few papers. Quality assessment by each review is shown in Appendix Tables 9 and 10.

### Quantitative findings

**Fig. 2:**
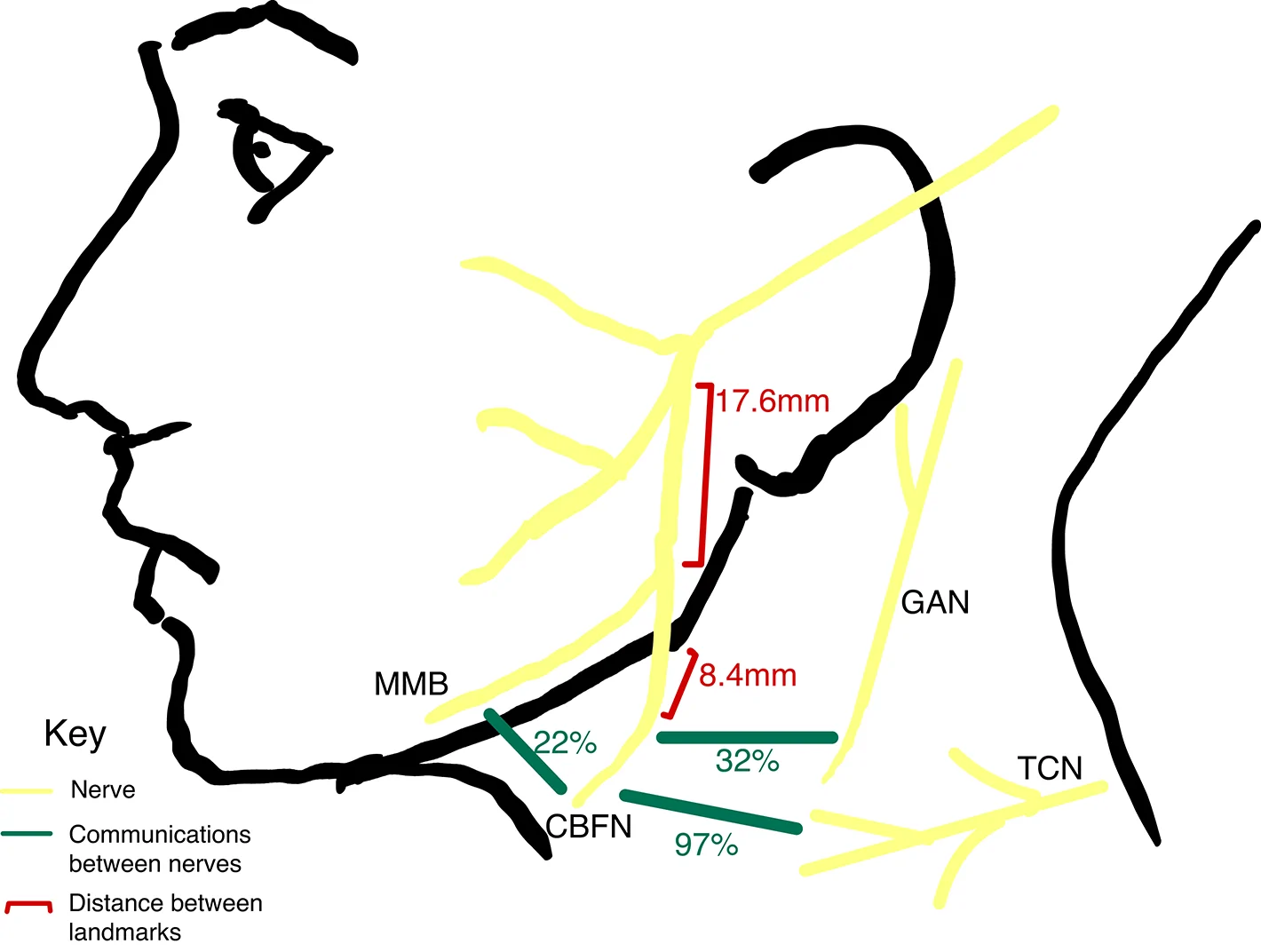
Summary of key quantitative findings

Commonly reported quantitative data included the number of branches of CBFN in each hemi-neck, distances of the CBFN from anatomical landmarks, communication prevalence between other nearby nerves, and the number of CBFN branches innervating the platysma. Figure 2 shows a summary of the key quantitative findings.

*22% prevalence of communications between CBFN and MMB; 32% prevalence of communications between CBFN and GAN; 97% prevalence of communications between CBFN and TCN; mean distance of 17.6 mm from the division of facial nerve trunk to the separation point of MMB and CBFN; mean distance of 8.4 mm from the angle of the mandible to CBFN. CBFN* = *Cervical branch of the facial nerve; GAN* = *Greater auricular nerve; MMB* = *Marginal mandibular branch; TCN* = *Transverse cervical nerve.*

#### Branching

A total of 11 papers reported individual CBFN branching frequency with numbers of branches ranging from one to three or more. Of these, only one paper reported individual frequencies for three or more branches (i.e. 3, 4, 5 branches). For consistency across studies, the higher-order branches reported in this paper were collapsed into the ≥ 3 category. Using the detailed counts provided in this paper, the mean number of branches within this ≥ 3 group was 3.5. This value of 3.5 was therefore used as the representative number of branches for the ≥ 3 category when calculating the pooled mean across all studies.

Across the 11 papers, a total of 346 specimens were included for analysis. Of these, 217 specimens (63%) had a single branch, 104 (30%) had two and 25 (7%) had three or more. The pooled mean number of cervical branch divisions was 1.34 branches per specimen, with a sensitivity range of 1.31 to 1.36 based on alternative assumptions for the ≥ 3 category, including a conservative assignment of three branches and an upper-bound scenario of four branches. The median and modal branch count were both 1.0. This is summarised in Table 3.

**Table 3:** Number of cervical branches of the facial nerve (CBFN) per hemi-neck

Author, Year	Hemi-necks, n =	No. of CBFN branches		
		1	2	3 +
Al Shaikh et al. [2]	20	8	12	0
Babuci et al. [5]	75	46	21	8*
Cakmak et al. [7]	20	2	18	0
Lindsey et al. [30]	15	7	6	2
Martinez Pascual et al. [32]	38	36	1	1
Minelli et al. [33]	12	0	5	7
Salinas et al. [38]	20	3	11	6
Sinno et al. [40]	16	16	0	0
Socolovsky et al. [43]	10	4	5	1
Tzafetta et al. [47]	10	7	3	0
Ziarah et al. [50]	110	88	22	0
Collated totals	346	217 (63%)	104 (30%)	25 (7%)

^*^Babuci et al. 2024 was the only paper to report individual frequencies for ≥ 3 branches, with 5 specimens having 3 branches, 2 specimens having 4 branches, and 1 specimen having 5 branches; the mean of these (3.5) was used as representative for the ≥ 3 category in pooled calculations

An additional two studies reported no individual frequencies but instead presented data in pooled ranges (e.g., “1–3 branches”) and therefore could not reliably be included in the analysis [36, 46].

#### Communications

A total of 10 papers with 432 hemi-necks reported 94 cases (22%; range of study means 3–70%) of communication of the CBFN with the MMB. A further two papers with a total of 95 specimens reported 30 cases (32%, range of study means 0.5–39%) of communication of the CBFN with the great auricular nerve (GAN). Finally, four papers with a total of 149 specimens reported 145 cases (97%, range of study means 80–100%) of communication of the CBFN with the transverse cervical nerve (TCN). This data is summarised in Table 4 and shown in Fig. 2.

**Table 4:** Number of hemi-necks with communications between the cervical branch of the facial nerve (CBFN) and adjacent nerves: the marginal mandibular branch of the facial nerve (MMB), transverse cervical nerve (TCN), and great auricular nerve (GAN)

Author, year	Hemi-necks, n =	CBFN-MMB, n (%)	CBFN-TCN, n (%)	CBFN-GAN, n (%)
Babuci et al. [5]	75	18 (24%)	75 (100%)	29 (39%)
Cakmak et al. [7]	20	14 (70%)	16 (80%)	NR
De Bonnecaze et al. [12]	30	1 (3%)	NR	NR
Domet et al. [13]	22	NR	22 (100%)	NR
Farahvash et al. [16]	42	5 (12%)	NR	NR
Freed et al. [17]	40	22 (55%)	NR	NR
MartinezPascual et al. [32]	38	2 (5%)	NR	NR
Minelli et al. [33]	12	3 (25%)	12 (100%)	NR
Salinas et al. [38]	20	6 (30%)	20 (100%)	1 (< 1%)
Woltmann et al. [49]	45	10 (22%)	NR	NR
Ziarah et al. [51]	110	13 (12%)	NR	NR
Collated means and totals		94 in 432 (22%)	145 in 149 (97%)	30 in 95(32%)

Sirasanagandla et al. (2012 and 2013) reported two separately published case reports in which the CBFN communicated with the TCN and GAN [41, 42], respectively, but as these were case reports, they could not be included in pooled statistics as they offered no information on prevalence.

#### Distances to anatomical landmarks

The mean shortest distance between the CBFN branching point and the AoM was reported by five papers with a total sample size of 112 specimens, and the pooled mean distance across the five papers was calculated as 8.4 mm (range of study means 7.5–10 mm).

A further five studies with a total of 96 specimens outlined the length of the facial nerve trunk (FNT) prior to bifurcation into the marginal mandibular and cervical branches, and the pooled mean distance of the FNT across the five papers was calculated as 17.6 mm (range of study means 13.0–27.9 mm) with wide variability across papers despite consistent methodology. This is illustrated in Tables 5 and 6, respectively and shown in Fig. 2.

**Table 5:** The shortest distance from the cervical branch of the facial nerve (CBFN) to the angle of the mandible (AoM)

Author, Year	Hemi-necks, n =	Distance from CBFN to AoM (mm)
Cakmak et al. [7]	20	8.6
Hontanilla et al. [22]	12	10
Salinas et al. [38]	20	7.5
Socolovsky et al. [43]	10	8
Ziarah et al. [50]	50	8.3
Collated total and mean	112	8.4

**Table 6:** The distance from the facial nerve trunk (FNT) to the point at which the cervical branch of the facial nerve (CBFN) and marginal mandibular branch (MMB) of the facial nerve separate

Author, Year	Hemi-necks, n =	Distance from FNT to CBFN-MMB separation (mm)
Chowdhry et al. [9]	16	17.5
Hontanilla et al. [22]	12	15
Martinez Pascual et al. [32]	38	14.4
Salinas et al. [38]	20	27.9
Socolovsky et al. [43]	10	13
Collated total and mean	96	17.6

#### Platysma innervation

The mean number of branches of the CBFN innervating the platysma was reported by three papers with a total sample size of 38, as shown in Table 7. The pooled mean number of branches of the CBFN innervating the platysma was 1.5 (range of study means 1–2).

**Table 7:** Mean number of cervical branches of the facial nerve (CBFN) innervating the platysma

Author, Year	Hemi-necks, n =	Branches
Hontanilla et al. [22]	12	2
Sinno et al. [40]	16	1
Socolovsky et al. [43]	10	1.7
Collated total and mean	38	1.5

#### Innervation of the depressor of the lower lip

Two studies (n = 16) reported on the prevalence of CBFN innervation of the depressor of the lower lip, with four out of 16 cases (25%, range of study means 20–33%) demonstrating such innervation: Salinas et al. (2009) found that in two out of six hemi-necks, CBFN innervated DLI [38], and Socolovsky et al. (2008) found this to be the case in two out of 10 hemi-necks [43].

### Observational findings

Qualitative descriptions of CBFN variation and outcomes reported by individual papers and case reports were thematically analysed.

#### Origin at parotid

Two papers commented on CBFN divisions in relation to the parotid gland: Salinas et al. (2009) noted that in 20 hemi-necks, 16 (80%) CBFNs divided before exiting, one (5%) separated at the exit point, and three (15%) separated after exiting the parotid gland [38]; Ziarah et al. (1981a) noted that in 110 hemi-necks, 88 (80%) had one branch at the parotid, and 22 (20%) had two branches [50].

#### Plane

Three papers described the CBFN as being in the plane deep to the platysma: Salinas et al. (2009) and Ziarah et al. (1981a) both described the CBFN as being anteroinferiorly deep to the platysma, superficial to the investing layer of deep cervical fascia [38, 50]. Socolovsky et al. (2008) noted that all of 10 (100%) cervical branches perforated the deep cervical fascia, deep to the platysma and superficial to the retromandibular and facial veins [43].

#### Communications

Freed et al. (2022) observed a single case of communication between the buccal and cervical branches in 40 hemi-necks (2.5%) [17]. Gerard et al. (2021) reported a case in which a duplicated TCN ran medial to a duplicated external jugular vein and communicated with the CBFN [18].

#### Relationship to anatomical landmarks

Two papers reported the relationship between CBFN and the mandible: Salinas et al. (2009) noted that all 20 (100%) branches were posterior to the AoM [38]; and Babuci et al. (2024) noted that, in four (7%) of 75 hemi-faces, the CBFN was found superior to the mandibular margin [5].

Gulati et al. (2024) noted that in a case where a branch of CBFN communicated with inferior loop of ansa cervicalis, this branch crossed 6 mm anterior to the AoM [20]. Ziarah et al. (1981a) noted that all 110 CBFNs were separate from MMB at the AoM and remained above the hyoid [50]. Sinno et al. (2019) described the CBFN as running below the thyroid cartilage [40].

Malins et al. (2022) dissected 11 hemi-necks to define a safe corridor for surgery between the sternocleidomastoid and the CBFN. The study established average distances between the CBFN and the anterior border of the sternocleidomastoid muscle, with the narrowest point averaging 8.8 mm [31].

Rossel Perry et al. (2016) identified a safe triangle consisting of points located at the lateral commissure of the mouth, the anterior border of the extracellular matrix muscle and the mastoid apophysis, in which the CBFN could be reliably located within a dissection of 64 hemi-faces [37].

#### Innervation

Arakawa et al. (2008) reported a case in which the platysma muscle was abnormally innervated by the glossopharyngeal nerve rather than the CBFN [4]. In this case, the CBFN maintained its function and course. This innervation was a consequence of an abnormal communication of the supernumerary branch of the glossopharyngeal nerve with the superficial cervical ansa. A common stem was formed between this branch of the glossopharyngeal nerve and a further anomalous branch from the external carotid plexus, which entered the cervical ansa alongside the CBFN.

Jovanovski et al. (2020) noted a case in which the GAN innervated the platysma in addition to the CBFN [25]. No causal factors were determined, but the GAN was observed to communicate with the CBFN whilst entering the deep surface of the platysma muscle. Four papers identified the CBFN innervating the depressor of the lower lip. Socolovsky et al. (2008) found that 20% of CBFNs innervated the depressor of the lower lip [43], and Salinas et al. (2009) described that one out of six CBFNs went through the platysma to innervate the depressor of the lower lip, and one out of six communicated with the MMB, and then innervated the depressor of the lower lip through the common branch [38]. Kaufman-Goldberg et al. (2023) noted that in 59 of 60 hemi-necks, lower lip depression was observed on stimulation of a distinct, inferior branch of the CBFN [27]. These findings were supported in a case report by Karakousis et al. (2000) in which the CBFN was sacrificed during neck dissection for malignant melanoma [26]. This led to a lack of eversion and caudal retraction of the lower lip during smiling despite preservation of the marginal mandibular nerve. Additionally, Klostermann et al. (2018) presented a case report of two sisters in whom the absence of CBFN led to palsy of the depressor of the lower lip [28].

#### Other observations

Eaton et al. (2019) reported a case in which one (1.4%) submandibular gland was pierced by the CBFN [14]. A further two papers noted variation in the relationship of the CBFN with surrounding vasculature: Cvetko et al. (2013) reported a single case in which the CBFN passed through a large fenestration in the external jugular vein [10] whilst Babuci et al. (2024) noted that in two (2.7%) hemi-necks, the CBFN had formed connecting loops around the retromandibular vein [5].

Leong et al. (2021) noted a variation in the course of the CBFN in which an aberrant CBFN arose proximally to the main cervicofacial nerve trunk’s bifurcation prior to platysma innervation [29]. As a result of this, the cervicofacial division did not cause platysma contraction when stimulated.

## Discussion

In the context of little primary anatomical data about the CBFN, poor awareness of the anatomy and its variations leaves patients vulnerable to iatrogenic injury and negative post-operative outcomes, both functional and aesthetic. Collating the existing data has allowed verification of the reliability of textbook descriptions and added further detail. Synthesis of primary anatomical data of the CBFN revealed variation in communication between nerves and a deeper understanding of the CBFN’s motor function. These findings hold important bearing, particularly for surgery involving the lower face and neck, diagnosis of CBFN injury, and iatrogenic damage prevention.

The CBFN displayed wide variability in its branching patterns, with reported branch frequency ranging from 1–3 + branches. Although Gray’s Anatomy specifically states that there is typically only one CBFN branch [45], this review revealed that this anatomical variant was present in only 63% of individuals. This mismatch in anatomical understanding may explain the common iatrogenic injury and misidentification of the facial nerve and its branches during head and neck surgery [44]. Additionally, the GAN (32%), marginal mandibular nerve (22%) and TCN (97%) showed a significant incidence of communication with the CBFN. While communication (motor) between the CBFN and other nerves has been widely reported, there was significant variation in prevalence, and this difference warrants further research. Such communications were mostly located posterior to or alongside the inferior border of the submandibular gland [13]. Submandibular gland excision has been noted as a high-risk procedure for facial nerve injury, with Brenan et al. (2019) also suggesting risk to the MMB due to limited operative exposure [6]. Taken together, careful appreciation of variable branching patterns of the CBFN and understanding of common areas of communication during surgical dissection are essential to minimise the risk of facial nerve injury during high-risk procedures. The frequent presence of multiple CBFN branches and communications may help explain why motor deficits following CBFN injury are often transient and are met with high rates of restoration of function.

Our findings show that the CBFN innervates the platysma with a mean of 1.5 branches across 38 specimens, challenging the view that a single CBFN branch is the typical anatomical variant for platysma innervation, as double-branch innervation was found to be equally common. This holds important implications for platysma dissection, as awareness of the high frequency of a second innervating CBFN branch may prevent inadvertent denervation during subplatysmal flap elevation or lower facial reconstructive procedures, where failure to preserve both branches could result in partial platysma paralysis or asymmetry [33]. The high prevalence of multiple branches of CBFN innervating the platysma is clinically significant in the context of injury to other nerves, such as the MMB. Since iatrogenic injury more commonly occurs to the MMB than CBFN, the CBFN has a significant role as a potential donor in nerve transfer [19], owing to its anatomical proximity. An added advantage is that CBFN and MMB innervate synergistic muscles, thus reducing the need for “central re-learning” and allowing for a faster postoperative recovery [36]. Thus, awareness of the common prevalence of multiple CBFN branches may inform intraoperative decision-making, particularly in the context of managing injury to nearby nerves.

Further motor function of the CBFN was identified throughout the literature as innervation of the DLI. Though its significance in the function of the lower lip was questioned [38], the role of the CBFN as the innervator of DLI was widely described. Across two papers, four (25%) patients were observed to have direct innervation of the lower lip depressor through the CBFN [38, 43]. The phenomenon of marginal mandibular pseudo-paralysis, described by Ellenbogen et al. [15], where post-operative inability to retrude the corner of the mouth was suggested to be due to injury to CBFN instead of MMB injury, could stem from this, though this was a contested phenomenon. Lindsey et al. (2023) acknowledged the ongoing debate regarding the innervation of the lower lip depressors, with their findings supporting the view that CBFN has a substantial contribution to lower lip function [30]. This was supported by the case reports by Karakousis et al. (2000) and Klostermann et al. (2018), where the absence of CBFN resulted in complete palsy of the lower lip depressor [26, 28]. Therefore, this review suggests that the CBFN serves a greater function than simply innervating the platysma, as is more commonly reported.

Various strategies for minimising iatrogenic injury to CBFN were uncovered by this review. A “safe surgical zone” for operative procedures involving the parotid and submandibular regions, including facelift surgery, was proposed by Lindsey et al. [30]. They recommended that continuous superficial musculoaponeurotic system-platysma dissection across the mandibular border into the neck can be performed safely when limited to the area proximal to the “cervical line”, which was defined as a line from the point 5 cm below the mandibular angle at the anterior border of the sternocleidomastoid muscle to the point where the facial vessels cross the mandibular border [30]. A “safe corridor” for surgery between the sternocleidomastoid and CBFN was also described [31]. Further methods suggested for mitigating the risk of iatrogenic CBFN injury included avoidance of “danger zones” such as the parotid gland [5], and preoperative mapping, though over-reliance on surface anatomy was cautioned due to the significant variability in CBFN course [5]. However, the results of this review highlight the potential importance of preoperative imaging and/or mapping rather than sole reliance on anatomical landmarks. The CBFN was associated with inconsistent distances from anatomical landmarks, with study mean distances differing by over two-fold in certain measurements. The need for caution when relying on surgical landmarks was highlighted by studies examining the variability of the facial nerve and its branches relative to nearby structures, such as the AoM and the temporomandibular joint [24, 39]. Whilst Sharma et al. (2023) described no significant differences in the distance of the facial nerve and its branches from anatomical landmarks across patients of varying stature [39], Ji et al. (2018) concluded substantial variations and cautioned the reliance on individual landmarks to guide surgery [24]. Our findings indicate that mean facial nerve trunk lengths prior to bifurcation into the CBFN and marginal mandibular nerve varied from 13 to 27.9 mm, highlighting this issue and stressing the importance of preoperative mapping.

Intraoperatively, nerve stimulation was suggested to correctly identify and subsequently protect the CBFN [43]. Extensive communications between nerves could mean that electrical stimulation of a given nerve could result in stimulation of others in its proximity, or of its target muscles. This could lead to intraoperative mislabelling of nerves; for instance, it has been reported that stimulation of communicating nerves of the cervical plexus elicited contraction of the platysma [41], despite the platysma being innervated by CBFN. This cautions against overreliance on intraoperative stimulation in the identification of nerves, and is supported by Leong et al. (2021), who reported a variation where the CBFN arose proximal to the main cervicofacial nerve trunk’s bifurcation and did not elicit platysma contraction when stimulated [29]. However, in combination with anatomical awareness and preoperative precautions of mapping the nerve using surface anatomy, these measures could reduce the risk and improve surgical accuracy.

A variety of observational findings of unique anatomical variations of the CBFN were identified through a thematic analysis of case reports. Common themes included variability in innervation patterns, communication and relationships to anatomical landmarks. By presenting a comprehensive overview of the diverse configurations reported in the literature, this summary serves as a practical reference point for surgeons, anatomists, and radiologists encountering atypical presentations. The broad variation in such presentations reinforces the necessity of individualised anatomical assessment rather than reliance on “typical” textbook descriptions.

While this review consisted of papers across five continents, with more than 800 collated specimens, it had some limitations, such as the limited geographical spread of the studies and methodological inconsistency. Despite no geographical limitations in the selection criteria, the vast majority, 17 out of 25 of the cadaveric studies were conducted in Europe, Australia, or the USA, accounting for 576 (70%) of 827 hemi-necks. While it may be argued that limiting the review to non-English studies could indirectly pose a geographic limitation in the selection criteria, our selection process identified only three papers which were excluded due to their language. The majority of included papers were from specimens from white ethnic backgrounds, thereby potentially limiting the generalisability of our findings. The quality of these studies was limited, with ambiguous definitions of measurements and insufficient methodological detail. Further quantifiable measures, such as the diameter of the CBFN, the number of fascicles, and measurements from consistent landmarks, were not reliably reported in the existing literature. Future studies would also benefit from finer methodological detail in the reporting of these cadaveric studies, including the competency or qualifications of dissectors, as well as the presence of two dissectors to corroborate findings, though it is understandable that in practice, this may be difficult to achieve. Specifying a core outcome checklist for dissectors to aid their reporting would enable consistent reporting across studies. The fact remains that very little research has been conducted into the anatomy of the CBFN, with only 35 relevant papers in over 43 years, of which only 25 were cadaveric/in-vivo studies. The inclusion of 10 case reports, while useful for supplementing or corroborating observations from the cadaveric studies, could not be reliably incorporated in the collated analysis. Further reports from in vivo dissections would also supplement data gathered from cadaveric studies and, combined with data on surgical outcomes and postoperative complications, could help form a wider picture of the clinical significance of protecting the CBFN during surgery.

## Conclusion

The review has expanded existing textbook knowledge of the cervical branch of the facial nerve by identifying the prevalence of communication with nearby nerves, variation in branching patterns, and the role played by CBFN in motor innervation of lower lip depressors. Awareness of anatomical variation identified in this review, combined with preoperative imaging and mapping, and consideration of intraoperative safe zones, can aid identification and preservation of CBFN, thereby reducing the risk of iatrogenic injury.

## Data Availability

The datasets generated and/or analysed during this study are available from the corresponding author on reasonable request.

## Appendix

See Appendix Tables 8, 9 and 10.

**Table 8:** Data extraction proforma

Year
Title
DOI
Journal
Country
Study design
Cadaveric versus Human
Number, n =
Left:Right side
Male:Female
Mean age
Ethnicity/nationality
Normal/syndrome
Reason for dissection
Deviation from normal anatomy
Prevalence of anomaly

**Table 9:** Quality assessment of cadaveric studies

Author	Reviewer A rating (12)	Reviewer B rating (12)
Al-Shaikh et al. 2015	6	6
Babuci et al. 2024	9	9
Cakmak et al. 2024	10	10
Chowdhry et al. 2010	6	6
De Bonnecaze et al. 2019	10	11
Domet et al. 2005	7	8
Eaton et al. 2019	9	9
Farahvash et al. 2013	9	9
Freed et al. 2022	9	9
Hontanilla et al. 2014	10	10
Kaufman-Goldberg et al. 2023	9	9
Lindsey et al. 2023	7	7
Malins et al. 2022	10	10
Martinez Pascal et al. 2019	9	9
Minelli et al. 2023	9	9
Rodriguez-Lorenzo et al. 2016	9	11
Rossell-Perry et al. 2016	7	7
Salinas et al. 2009	8	8
Sinno et al. 2019	9	9
Scolovsky et al. 2008	7	7
Suganya et al. 2022	9	9
Tzafetta et al. 2007	11	12
Woltmann et al. 2006	7	7
Ziarah et al. 1981a	8	8
Ziarah et al. 1981b	11	11

QUACS analysis summary table—Primary cadaveric studies, out of a total score of 13

**Table 10:** Quality assessment of case reports/series

Author, year	Reviewer A rating (8)	Reviewer B rating (8)
Arakawa et al. 2008	3	3
Cvetko et al. 2013	3	3
Gerard et al. 2021	4	3
Gulati et al. 2023	3	3
Jovanovski et al. 2020	1	2
Karakousis et al. 2000	7	8
Klosterman et al. 2018	7	7
Leong et al. 2021	8	7
Sirasanagandla et al. 2012	3	3
Sirasanagandla et al. 2013	3	2

Case report quality assessment, out of a total score of 8
